# The Yellow Fever Outbreak in Brazil (2016–2018): How a Low Vaccination Coverage Can Contribute to Emerging Disease Outbreaks

**DOI:** 10.3390/microorganisms13061287

**Published:** 2025-05-31

**Authors:** Juliane Duarte Santos, Kamila Lorene Soares Rocha, Carolina Dourado Amaral, Ana Gabriella Stoffella Dutra, Poliana de Oliveira Figueiredo, Etel Rocha-Vieira, Filipe Vieira Santos de Abreu, Giliane de Souza Trindade, Danilo Bretas de Oliveira

**Affiliations:** 1 Laboratório de Doenças Infecciosas e Parasitárias, Universidade Federal dos Vales do Jequitinhonha e Mucuri, Diamantina 39100-000, Minas Gerais, Brazil; juliane.santos@ufvjm.edu.br (J.D.S.); etel.vieira@ufvjm.edu.br (E.R.-V.); 2 Laboratório de Vírus, Instituto de Ciências Biológicas, Departamento de Microbiologia, Universidade Federal de Minas Gerais, Belo Horizonte 31270-901, Minas Gerais, Brazil; kamilalsr@yahoo.com.br (K.L.S.R.); anagstoffella@gmail.com (A.G.S.D.); gitrindade@yahoo.com.br (G.d.S.T.); 3 Núcleo de Inovação em Doenças Infecciosas Emergentes e Re-Emergentes, Secretaria de Estado de Saúde de Minas Gerais, Belo Horizonte 31630-903, Minas Gerais, Brazil; carolinadamaral@gmail.com; 4 Superintendência de Desenvolvimento da Capital—SUDECAP, Belo Horizonte 30180-105, Minas Gerais, Brazil; polianaofigueiredo@yahoo.com.br; 5 Instituto Federal do Norte de Minas Gerais, Campus Salinas, Salinas 39560-000, Minas Gerais, Brazil; filipe.vieira@ifnmg.edu.br

**Keywords:** *Orthoflavivirus flavi*, epidemiology, Brazil, emerging disease

## Abstract

Yellow fever (YF) disease is a viral infection caused by *Orthoflavivirus flavi* (YFV). YFV is transmitted by hematophagous daytime-biting mosquitoes, predominantly *Haemagogus* spp. and *Sabethes* spp. in the sylvatic cycles, and *Aedes* spp. in urban cycles. In this work, we correlated vaccination coverage with the occurrence and spread of the disease throughout Brazil during the years 2016–2018. The Vale do Mucuri and Vale do Rio Doce regions in Minas Gerais state had the highest number of reported cases. Despite being considered areas with vaccine recommendation since 2008, these regions had less than 60% and 70% vaccination coverage in 2016. The outbreak of YF in Brazil has shown that surveillance for emerging diseases should be constant, especially in relation to the national immunization program. In this study, we observed that low vaccination coverage and the lack of public policies aimed at this region with low population development may have an impact on the reemergence of YF.

## 1. Introduction

The yellow fever (YF) disease is a classic viral hemorrhagic fever (VHF), with pansystemic viral infection [1]. The disease can present a variety of clinical manifestations, including fever, myalgia, lumbar pain, headache, nausea, and vomiting. Symptoms typically appear within the first three days of illness following an incubation period of 3–6 days after infection via a mosquito bite [1,2]. In some cases, after a 24 to 48 h period of symptom remission, the disease recurs with heightened severity, leading to renal insufficiency, jaundice, recurrent fever, and hemorrhagic manifestations [1,2]. The case-fatality rate during this phase ranges from 20% to 25% [1,2].

The *Orthoflavivirus flavi* (YFV) was the first pathogen of the *Orthoflavivirus* genus, Flaviviridae family, to be isolated from a human host [3,4], with a constant occurrence in Africa and South America [1]. Molecular evolutionary analysis estimates that YFV and *Orthoflavivirus denguei* diverged from a common ancestor approximately 3000 years ago, although the first documented record of YF appears in a 1648 Mayan manuscript, with reports of epidemics occurring in Mexico and Guadeloupe during that same year [1,4,5]. It is presumed that the YFV originated in Africa and expanded worldwide in the 16th century [6,7]. This virus caused epidemics and outbreaks in Africa, Europe, and North and South America, and it is an endemic disease in the tropical areas of Africa and South America [1,5,8].

YFV is maintained in nature through three distinct transmission cycles [1,5,9]. The urban cycle, involving human–vector–human transmission through the bite of the *Aedes aegypti* mosquitoes, has not been reported in Brazil since the 1940s [1,5]. The sylvatic cycle includes transmission between the non-human primates and sylvatic mosquitoes, such as *Haemagogus* spp. and *Sabethes* spp. in the Americas, and *Aedes* spp. in Africa; however, humans may become accidentally infected when entering sylvatic habitats [1,5]. In Africa, an additional intermediate cycle occurs through semi-domestic mosquitoes that transmit the virus between humans and non-human primates in forest-border areas [9].

## 2. Yellow Fever Vaccine

The early 1900s were a milestone in YF research, when Walter Reed and his commission demonstrated that disease transmission was associated with a vector mosquito, and that the etiological agent could pass through bacteriological filters, suggesting a non-bacterial pathogen [10,11]. This hypothesis was confirmed in 1927 when researchers Adrian Stokes, Bauer, and Hudson first isolated a YFV strain from human blood (*Asibi*) [11]. Their experimental results demonstrated the viral etiology of YF, showing that rhesus monkeys (*Macaca mulatta*) developed the disease following inoculation with infected human blood, thus confirming the interspecies transmissibility of the viral agent [11].

Later, between 1935–1937, Max Theiler and collaborators conducted a series of experiments using the *Asibi* strain of the YFV [7,11]. Through repeated serial passages of the virus in minced chick embryos whose central nervous system had previously been removed, they obtained a viral variant that remained genetically stable and exhibited no neurotropic effects, even after subsequent passages in chick embryo cultures containing brain tissue [7,11]. The attenuated viral variant derived from these passages was eventually designated the 17D strain [7,11]. In 1936, Theiler and his colleagues initiated clinical trials that confirmed the 17D strain’s ability to elicit robust and long-lasting immunity without inducing disease, leading to the development of the 17D live attenuated vaccine [7,8,12]. In 1937, the Rockefeller Foundation implemented the first large-scale field trial of the 17D YF vaccine in Brazil [7,11]. The results demonstrated high efficacy and safety in human populations, enabling the subsequent rollout of mass vaccination campaigns in Brazil and other endemic countries during the late 1930s, which led to a significant reduction in the incidence of YF [7,11].

In accordance with current World Health Organization (WHO) recommendations, all licensed YF vaccines must be manufactured exclusively from the live-attenuated 17D viral strain propagated in embryonated chicken eggs [7,13]. Furthermore, safety testing of the vaccines must be conducted exclusively in non-human primate models, such as rhesus and cynomolgus macaques [7,13]. Three substrains of 17D are used worldwide in vaccine production: 17DD, which is employed by the public health system in Brazil and produced domestically by Bio-Manguinhos/Fiocruz; 17D-204, manufactured in France, Senegal, the United States, and China; and 17D-213, derived from 17D-204 and produced in Russia [12,13,14,15,16]. Vaccines manufactured in France, Russia, Senegal, and Brazil have undergone the WHO prequalification process, ensuring their adherence to international quality standards and enabling their use in global vaccination campaigns [16]. The vaccines produced in the United States and China are primarily intended for domestic use, but this does not imply inferior quality compared to WHO prequalified vaccines, as not all manufacturers choose to submit their products for prequalification [13,16].

YF vaccine induces effective immunity in 80–100% of vaccinated individuals within 10 days, and in over 99% within 30 days post-vaccination [1,2]. In Brazil, a study conducted in the city of Recife evaluated the immune response of 238 individuals after receiving the first dose of the 17DD YF vaccine [17]. All participants demonstrated seroconversion as detected by the plaque reduction neutralization test (PRNT) [17]. More than 30 days post-vaccination, 70.6% had detectable anti-YFV IgM antibodies and 98.3% had IgG antibodies, indicating a strong immune response induced by the vaccine [17]. A single dose is sufficient to confer lifelong immunity and protection, with no need for booster doses [2,18]. Long-term immunogenicity has been widely demonstrated, as in a follow-up study of U.S. military veterans from World War II, in which over 80% retained neutralizing antibodies 30–35 years after a single dose of the vaccine, with titers exceeding 95% in certain subgroups [19]. Although studies confirm the YF vaccine’s long-term immunogenicity, population-based variations in immune responses have been documented [7,20]. A clinical trial (n = 1440) comparing two licensed vaccines (YF-VAX and Arilvax) revealed significantly higher neutralizing antibody titers in male and Caucasian participants compared to female, Black, and Hispanic individuals for both vaccines [7,20].

Whereas adverse effects associated with the YF vaccine are rare, its administration is contraindicated in some conditions [17]. This include infants under 9 months of age, pregnant women (except during outbreaks when infection risk outweighs vaccine risks), individuals with severe hypersensitivity to egg proteins, and persons with significant immunodeficiency, such as those with symptomatic HIV/AIDS or other causes of immunosuppression, as well as individuals with thymic disorders [1,2,7,15]. In rare cases, serious adverse events may occur following YF vaccination. Mild or self-limiting adverse events were observed in approximately 25% of vaccinated individuals, with symptoms such as headache, low-grade fever, discomfort at the injection site, muscle pain, and headache [6,7,21].

YF vaccine–associated viscerotropic disease (YEL-AVD); YF vaccine–associated neurotropic disease (YEL-AND); and anaphylaxis are the most concerning adverse effects of YF vaccine [21,22]. YEL-AVD, which closely resembles wild-type YF, occurs in approximately 0.3 per 100,000 vaccinations and may lead to multi-organ failure and death [21,22]. Early symptoms typically appear within one week of vaccination and include fever and other nonspecific manifestations such as headache, malaise, myalgia, nausea, vomiting, or diarrhea. As the condition progresses, patients may develop jaundice and laboratory abnormalities including thrombocytopenia, elevated hepatic transaminases, increased total bilirubin, and elevated serum creatinine [21]. Leukopenia or leukocytosis may be present, depending on the phase and severity of the illness. In severe cases, complications such as hypotension, hemorrhage, renal failure requiring hemodialysis, and respiratory failure necessitating mechanical ventilation may arise [21]. Less commonly, rhabdomyolysis and disseminated intravascular coagulation have been reported. Management is primarily supportive [6,7,21,22].

YEL-AND, although rarely fatal, occurs in about 0.4 per 100,000 vaccinations and is characterized by neurological complications such as meningoencephalitis and acute disseminated encephalomyelitis [21]. These manifestations result either from direct viral invasion of the central nervous system, affecting the meninges and/or brain, or from autoimmune mechanisms, where vaccine-induced antibodies and/or T cells cross-react with neuronal antigens, leading to central or peripheral nervous system damage. Treatment for YEL-AND is tailored to the specific clinical presentation [6,15,22].

Anaphylactic reactions to the YF vaccine, although rare, occur at an estimated rate of 0.8 cases per 100,000 vaccinations [21]. This severe allergic response, which involves multiple organ systems, is more prevalent in individuals with a history of egg allergies or sensitivity to specific vaccine components [23]. The 17D YF vaccine production employs infected chicken embryos and may contain residual egg or chicken proteins [7,12,24]. As a result, it is contraindicated in individuals with a known history of egg or chicken allergy due to the potential risk of hypersensitivity reactions [6,7,19]. Additionally, the vaccine contains gelatin, which is commonly used as a stabilizer in several vaccines. Gelatin has been implicated as a potential allergen and may contribute to allergic reactions in susceptible individuals. Therefore, careful assessment of allergy history is essential prior to administration of the YF vaccine [23,24].

Despite the rare adverse events associated with the 17D YF vaccine, large-scale immunization remains a fundamental prevention strategy [7,8]. High vaccination coverage substantially reduces the incidence of the disease, protecting both individuals and entire communities [7]. Given the constant risk of new YF outbreaks of varying magnitudes, the Pan American Health Organization (2023) recommends an optimal vaccination coverage equal to or greater than 95% [18].

Although the YFV vaccine was developed almost 100 years ago [7,22,25], recent outbreaks in Africa and South America, accompanied by increased cases in previously non-endemic areas, indicates that both the virus and its vector are spreading to regions with low or no vaccination coverage [26,27].

## 3. The Most Recent Outbreak in Brazil: From Minas Gerais State to the Country

In Brazil, YF arose at the end of the 17th century; thenceforth, this disease has become the most important epidemic disease in the country [28]. Between the 1970s–1990s, Brazil was responsible for 16% of YF notifications in Latin America, the largest number of YFV cases for this period in this region [29]. In 1999, the endemic area of YFV was localized in the north and midwest of Brazil, including the Amazonic and pre-Amazonic areas and the Maranhão State. Sporadic cases have been reported in the Minas Gerais State [29,30,31]. Between 2002 and 2008, the circulation of YFV also expanded to the east and south of the country [31]. Because of the large number of cases and their severity, YF became a disease of compulsory notification in Brazil after 2006 [31].

In 2014, the YFV was detected on the border between the northern and midwest of Brazil [32,33]. After this period, YFV spread to southeastern and southern Brazil, where the transmission was concentrated. In Minas Gerais, for example, epizooties were found in non-human primates in 2014 and 2015 [30,34]. Between 2016 and 2018, Brazil experienced the largest sylvatic YF outbreak, marked by intense transmission through the sylvatic cycle and resulting in a high number of infections and deaths among both humans and non-human primates since the 1980s [27,35,36,37]. This outbreak, which began in December 2016, spread beyond the endemic Amazon region and affected unvaccinated populations in rural areas across all four states of southeastern Brazil, highlighting the vulnerability of non-endemic regions to YF epizootics and epidemics. [27,38]

According to data from the Brazilian Ministry of Health, during the monitoring period, from 2016 to 2017, 3564 human YF cases were reported in Brazil (777 confirmed), with the highest number of cases in the country’s southeast [37,39]. Minas Gerais was the epicenter of the outbreak, with about 1695 cases (465 confirmed), followed by Espírito Santo, with 878 cases (252 confirmed), São Paulo with 432 cases (22 confirmed), and in the Rio de Janeiro State, 111 cases (25 confirmed) were described [39]. According to the probable site of infection, the cases were reported in 188 municipalities, of which 49.4% (379 cases) were in Minas Gerais, followed by Espírito Santo (93 cases), São Paulo (4 cases), and Rio de Janeiro (2 cases) [39]. In the following year, during the seasonal monitoring period from July 2017 to June 2018, an increase in case numbers was documented, with 5052 human cases (1127 confirmed) reported in Brazil [37,39]. Of these, 4498 cases (940 confirmed) were reported in the southeastern region: the São Paulo State reported the highest number of cases (2513 reported, 514 confirmed), followed by Minas Gerais (1444 reported, 320 confirmed), Rio de Janeiro (426 reported, 85 confirmed), and Espírito Santo (115 reported, 21 confirmed) [37,40].

**Figure 1:**
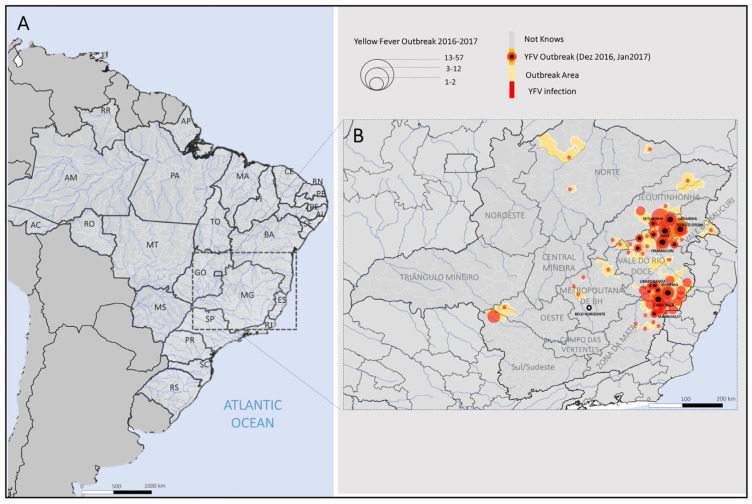
YF cases were reported in southeast Brazil in 2016–2017. (**A**). Minas Gerais State localization in Brazil (**B**). The circles show the number of cases in municipalities of Minas Gerais in 2016 and 2017. The black spots indicate the municipalities that presented cases in December 2016 and January 2017.

The outbreak was predominantly concentrated in Minas Gerais (Figure 1A) [39,41,42]. From December 2016 to January 2017, 107 confirmed human cases were reported across 16 municipalities, representing the first recorded outbreak in Brazil’s southeastern region [40,43]. Spatial analysis revealed simultaneous case emergence in multiple regions, with the highest burden in eastern Minas Gerais—notably the Jequitinhonha Valley, Rio Doce Valley, Mucuri Valley, and Zona da Mata districts (Figure 1B) [40,43].

## 4. The Viral Genotype Circulating in Brazil

YFV is a single-stranded, positive-sense RNA virus with an approximately 11 kb genome [38]. Although it presents only one serotype, distinct lineages have been identified, with observed strain variations correlating with geographical distribution [38]. Evidence indicates that YFV arose in Africa several millennia ago and was introduced in the Americas from West Africa approximately 300–400 years ago, most likely via the transatlantic slave trade [8,27].

African viruses were grouped into five main lineages: West Africa I, West Africa II, East Africa, East/Central Africa, and Angola. Most African strains were classified under WA-II, while WA-I had the fewest viral sequences identified [44]. YFV in the Americas is classified into two geographically distinct genotypes. Genotype I predominantly circulates in Brazil and several northern Latin American countries, including Trinidad, Venezuela, Ecuador, Panama, and Colombia [27,45,46]. Genotype II, on the other hand, is primarily found in Andean countries such as Peru and Bolivia but has also been identified in Brazil, Ecuador, and Trinidad [27,38].

During the mid-1990s, a substantial shift in the genetic diversity of YFV genotype I was observed, marked by the emergence of a new lineage, referred to as the “modern lineage” (1D and 1E) [27,32,35,38,47]. This modern lineage progressively replaced the previously circulating lineages (1A–1C) and has been associated with outbreaks in both endemic and non-endemic regions of South America throughout the 21st century [27,35].

Current evidence suggests that lineage 1E of YFV likely originated in northern Brazil circa 1989, with subsequent rapid dissemination of a sublineage (genetic variants of the virus that have spread and adapted in specific regions) from the north to the central-west region by 1993, reaching non-endemic regions (northeast, southeast, and south of the country) [27].

Genomic and epidemiological surveillance data from the recent outbreak indicate that the circulating virus shows closer phylogenetic relationships to strains previously identified in northern Brazil [27]. The 2016–2018 outbreak was caused by a variant from Amazonia, an endemic region, rather than from the re-emergence of a lineage that could have persisted in Minas Gerais state from the 2003 outbreak [27,30,48]. Rezende et al. reported that during the outbreak of 2016–2017, there was a single introduction of endemic YFV in the southeast, probably from the central-west region [32]. It was also shown that the same lineage was responsible for the cases detected in 2018, which indicates the persistence of the YFV outside the Amazon basin during this interepidemic period [32]. YFV genomes isolated from humans, non-human primates, and mosquitoes collected across five Brazilian states between 2015 and 2018 were also analyzed by Delatorre et al. [47]. The findings revealed the circulation of two distinct sub-lineages in the State of Goiás (central-west region). Both sub-lineages shared the molecular signature of the strains responsible for the outbreak in the southeast. Further, they followed independent routes of dissemination toward Minas Gerais, one advancing through the southeastern Atlantic basin to Espírito Santo and Rio de Janeiro, and the other spreading toward São Paulo via the Paraná basin [47]. A third study analyzed complete YFV genome sequences obtained in 2017 from Minas Gerais, Espírito Santo, and Rio de Janeiro [48]. It supports the hypothesis that the outbreak began in Minas Gerais, around July 2016, with subsequent southward spread to Rio de Janeiro and São Paulo, states where YF vaccination was not recommended until 2017 and 2018, respectively [48].

The spread of YFV beyond the Amazon and Cerrado biomes, following hydrographic basins that cross major urban centers, underscores the importance of genomic surveillance integrated with epidemiological and spatial data. This approach is essential to guide control strategies, expand vaccination coverage, and prevent urban transmission [32,47,48].

## 5. Low Vaccination Coverage: The Beginning of an Outbreak

The increase in YF cases reported in South America may be explained by multiple factors that are not entirely understood, such as climatic changes, higher urbanization rates, and expansion of the human population [8,29]. In addition, the low vaccination coverage in the regions with disease outbreaks, the possible presence of efficient sylvatic vectors in degraded peri-urban habitats, and the absence of vaccination certificates for travelers can contribute to disease resurgence [5,8,32,34].

**Figure 2:**
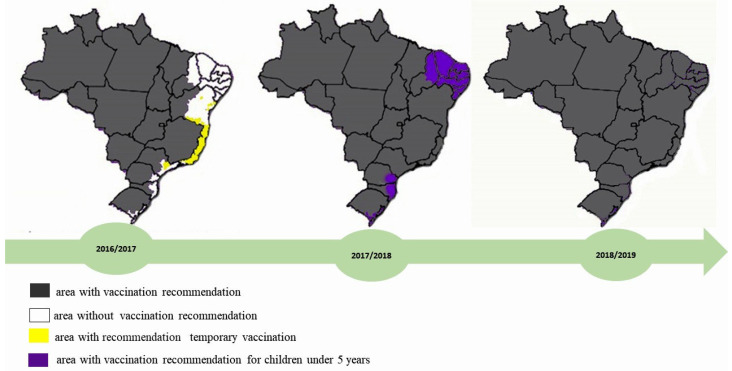
Brazilian areas with YFV vaccine recommendation. The areas with permanent vaccination recommendations are shown in gray. The areas in white are those without YFV vaccine recommendation. Finally, the yellow areas are those with temporary vaccination recommendations.

Between 2000 and 2008, the expansion of YFV circulation in Brazil prompted the Brazilian Ministry of Health to revise its risk area classification system [31]. The previous designations of endemic, transition, and indene areas were replaced with two new categories: vaccination recommended areas (ACRV), which are areas where there is a history of sylvatic YF, and vaccination not recommended areas (ASRV), which are indene areas [31,33,41,42]. The new classification considered the following variables: virological surveillance data, ecosystem characteristics (including watersheds and vegetation patterns), wildlife migration routes, human mobility patterns, illegal animal trade, and healthcare infrastructure organization [49]. During the 2016–2018 outbreak, the Brazilian Ministry of Health expanded YF vaccination recommendations to the entire national territory, including previously non-endemic areas [49,50]. This policy change responded to the virus’s geographic expansion into new regions that were formerly considered low risk, implementing preventive measures to establish population-wide protection in anticipation of a potential increase in viral spread [50]. The figure below presents a map of the Brazilian areas with YF vaccine recommendation in this period (Figure 2).

In response to the exponential rise in YF cases, the Brazilian Ministry of Health distributed 68.9 million vaccine doses nationwide between 2017 and 2018, 45.1 million in the first year and 23.8 million in the subsequent year [37]. Another important measure adopted by the Brazilian Ministry of Health and supported by the WHO was the administration of vaccine fractions in the States of Rio de Janeiro, São Paulo, and Bahia, in which one-fifth (1/5) of a standard dose (0.5 mL) was administered [37]. Because of viral circulation, the strategic use of fractionated doses can protect a larger proportion of the population while limiting the amount of vaccine necessary. This is the most extensive fractionated dose vaccination campaign ever conducted [18,37,49,50,51]. The immunogenicity and safety of fractional doses of four WHO-prequalified YF vaccines was previously reported, demonstrating high seroconversion rates (98.2% to 100%) at 28 days post-vaccination [52]. A study conducted in São Paulo evaluated viremia and immune responses induced by fractional-dose versus standard-dose YF vaccination. Fractional dose elicited a more complex immunological network involving follicular helper T cells and B cells compared to the standard dose. These findings support the feasibility of dose-sparing strategies in adults during outbreaks, providing an effective alternative to maximize vaccine coverage in settings with limited supply [52]. Further, these findings suggest that fractional dosing may serve as a viable alternative to the standard regimen [26].

**Figure 3:**
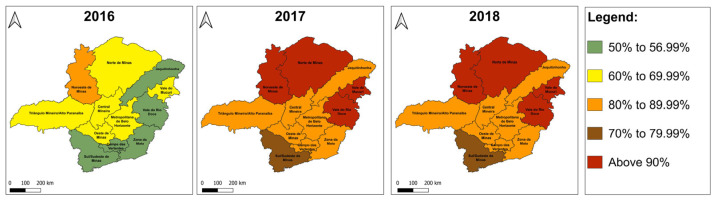
Temporal evolution of vaccine coverage in the regions of Minas Gerais state, between 2016 and 2018.

Since 2008 in Minas Gerais, the epicenter of the most recent outbreak, vaccination has been recommended in all 12 mesoregions, which have a high variance in vaccine coverage. In December 2016, primate deaths and probable cases of YF in humans were recorded in 4 Regional Health Units [36,37]. That same year, when the outbreak started, only 57.26% of the population was covered by vaccination. Moreover, only the northwest of the state, where YFV has been registered since 1999, had a vaccine coverage above 80% [36,37]. Up to July 2017, 186 (121 confirmed) municipalities had notified epizootics. On the contrary, the state’s vaccination coverage significantly increased to 82.97%. In the north, northwest, Vale do Mucuri, and Vale do Rio Doce macroregions, the vaccination coverage was superior to 90%, and only the south/southeast did not reach more than 80% coverage [37,39,40]. In 2018, epizooties were reported in 379 (86 confirmed) municipalities, when vaccination coverage reached 91.11%. However, in the south/southeast, vaccination remained below 80% (Figure 3) [37,39,40]. Currently, among the 853 municipalities of Minas Gerais, the vaccine coverage accumulated between 1997 and 2022 does not reach 80% in 49 cities, corresponding to 5.74% of the State. Another 5.04% (43) of the municipalities have between 80% and 94.99% of their population vaccinated, and 89.2% have more than 95% coverage (761). Thus, we observed a significant vaccination rate in the municipalities of Minas Gerais state [50].

The outbreak of YF in Brazil shows that surveillance for emerging diseases should be constant, especially regarding the national immunization program [32]. Overall, in endemic and epizootic areas of Brazil, YF is more prevalent among men aged 14 to 35 years, particularly among those who develop occupational activities carried out near forests (loggers, rubber tappers, rural workers, miners, hunters, Amazon riverside dwellers), which increases exposure to the sylvatic cycles of YFV transmission [1,53]. During the 2017–2018 transmission period in Brazil, most confirmed cases affected economically active male individuals with no prior vaccination history. Among these, rural residents were the most affected [37]. Therefore, the vaccination of these residents is essential for building a “belt” of vaccinated persons to prevent the entry of the virus in urban environments [28,53]. In several regions of Brazil, rural residents have limited access to the health system and vaccines, and so they must be targeted for immunization programs and campaigns against YFV [39]. In some states of Brazil, as in the case of Minas Gerais, vaccination coverage calculation is done by the ratio between applied doses and the number of individuals in the population; as such, the vaccination coverage rate in rural populations may be underestimated [54].

## 6. Lessons and Perspectives

The cases reported here demonstrate that an improvement in vaccination coverage is needed, and an emergency plan must be established to deal with outbreaks of infectious diseases. This includes laboratories prepared for the diagnosis in a short period of time and continued training of the clinical staff. This will allow the identification of different forms of clinical manifestation of YFV and fast treatment.

Molecular analyses of YFV samples from the last outbreak were of great importance for the elucidation of viral origin, excluding the possibility of the occurrence of cases due to vaccination reversal. In addition, by characterizing the viral genetic diversity and the specific genetics of the circulating virus, the adequacy of diagnostic tests and genetic markers of disease severity can be assessed.

## Data Availability

No new data were created or analyzed in this study.

## Abbreviations

The following abbreviations are used in this manuscript:

ACRV	Area with vaccine recommendation
ASVR	Area without vaccine recommendation
VHF	Viral hemorrhagic fever
WHO	World Health Organization
YF	Yellow fever
YFV	*Orthoflavivirus flavi*
